# Hospitalisation for Drug Infusion Did Not Increase Levels of Anxiety and the Risk of Disease Relapse in Patients with Inflammatory Bowel Disease during COVID-19 Outbreak

**DOI:** 10.3390/jcm10153270

**Published:** 2021-07-24

**Authors:** Lorenzo Bertani, Brigida Barberio, Domenico Tricò, Federico Zanzi, Daria Maniero, Linda Ceccarelli, Ilaria Marsilio, Francesca Coppini, Greta Lorenzon, Maria Gloria Mumolo, Fabiana Zingone, Francesco Costa, Edoardo Vincenzo Savarino

**Affiliations:** 1 Department of Translational Research and New Technologies in Medicine and Surgery, University of Pisa, 56100 Pisa, Italy; federico.zanzi@outlook.com (F.Z.); coppini.francesca@gmail.com (F.C.); 2 Department of Surgery, Tuscany North-West ASL, Massa Apuane Hospital, 54100 Massa, Italy; 3 Department of Surgery, Oncology and Gastroenterology, DISCOG, University of Padua, 35100 Padua, Italy; brigida.barberio@gmail.com (B.B.); daria.maniero@unipd.it (D.M.); ilaria.marsilio@gmail.com (I.M.); gretalorenzon90@gmail.com (G.L.); fabiana.zingone@unipd.it (F.Z.); edoardo.savarino@unipd.it (E.V.S.); 4 Department of Surgical, Medical and Molecular Pathology and Critical Care Medicine, University of Pisa, 56100 Pisa, Italy; domenico.trico@unipi.it; 5 Department of General Surgery and Gastroenterology, IBD Unit, Pisa University Hospital, 56124 Pisa, Italy; ceccarellilinda@gmail.com (L.C.); g.mumolo@int.med.unipi.it (M.G.M.); fcosta@med.unipi.it (F.C.)

**Keywords:** clinical, Crohn’s disease, ulcerative colitis, biologics, COVID-19

## Abstract

During the coronavirus disease 2019 (COVID-19) pandemic, immunomodulatory therapies and hospital admission were suspected to increase the risk of infection. Nevertheless, patients with inflammatory bowel diseases (IBD) treated with intravenous (i.v.) biologics had to move to hospitals for drug infusion. We investigated the impact of hospitalisation in patients with IBD. We conducted a survey including consecutive IBD patients initially in clinical and biochemical remission treated with biologics at the end of the first lockdown period. Patients underwent the normally scheduled clinical visits, performed at hospital for i.v.-treated patients or at home for patients treated with s.c. drugs. We administered to all patients the Hospital Anxiety and Depression Scale (HADS) questionnaire and other 12 questions, specifically related to COVID-19 and its implications. A total of 189 IBD patients were recruited, 112 (59.3%) treated with i.v. drugs and 77 (40.7%) with s.c. ones. No relapses were recorded in either group (hospitalized vs. non-hospitalized, *p* = ns), as well as which, COVID-19 infections were not demonstrated in patients in contact with people with suspected symptoms or directly experiencing them. The total HADS score obtained by the sum of all items was also almost identical between groups (37.1 ± 2.8 vs. 37.2 ± 2.8; *p* = 0.98). In patients treated with i.v. drugs receiving a televisit (*n* = 17), the rate of satisfaction with telemedicine (58.8%) was significantly lower compared with those treated with s.c. drugs (94.8%; *p* < 0.0005). Our results suggest that hospitalisation during the COVID-19 outbreak does not increase the risk of COVID-19 infection as well as the risk of IBD relapse; moreover, the similar levels of anxiety in both groups could confirm that there is no need to convert patients from i.v. to s.c. therapy.

## 1. Introduction

The coronavirus disease 2019 (COVID-19) pandemic has revolutionized our habits of life in the last 12 months. Presently, more than 115,000,000 people have been infected with severe acute respiratory syndrome coronavirus 2 (SARS-CoV-2), and more than 2,500,000 people have died because of it [1]. On 10 March 2020, at the beginning of the diffusion of the virus in Western countries, the Italian Government decided to limit the spread of SARS-CoV-2 by declaring a national lockdown. Home confinement exposed individuals to a stressful situation of unknown duration [2]. Moreover, broadcasters focalized their program schedules on COVID-19 pandemic, leading to an “infodemic”, which had significant impact on the psychological well-being of the whole population, especially with regard for the access to hospitals [3]. 

Inflammatory Bowel Diseases (IBD) are chronic disorders of the intestinal tract characterized by relapsing and remitting intestinal inflammation [4,5]. Both diseases are associated with a marked reduction in health-related quality of life, severe fatigue, and work impairment, as well as with depression and anxiety [6,7]. Indeed, several studies associated the risk of disease relapse to stressful events [7,8,9].

When standard treatments fail to achieve remission [10], patients with IBD are treated with biologic drugs, which have been associated with a reduction in immune response in case of infections. In particular, the use of anti-TNF drugs has been associated with an increased risk of viral and bacterial infections, including pneumonia [11,12,13]. Therefore, several patients with IBD were particularly worried about being more susceptible to SARS-CoV-2 infection [14]. Along the same lines, even the physicians shared this fear, since a European survey conducted in March 2020 in several European IBD centres showed that 67.7% of participants believed that immunomodulators and biological drugs could be a predisposing factor for SARS-CoV-2 infection [15].

The management of IBD patients has necessarily been modified during the most difficult phases of the pandemic, resetting the clinical priorities [16]. For instance, in one of the IBD centres in Wuhan, China, all patients treated with biologics and immunomodulators discontinued these therapies [17]. This was performed in order to avoid patients having to move to high-risk of transmission places such as hospitals or infusion centres [17]. However, the international guidelines recommended the continuation of the ongoing treatment, including biologics, in order to avoid disease relapses [18], initially suggesting converting i.v. therapies to s.c. drugs [19,20]. In our country, most of the IBD patients continued their ongoing treatment, performed in hospital in the case of intravenous (i.v.) drugs, whereas the patients treated with subcutaneous (s.c.) drugs could stay at home with control visits carried out with telemedicine [21,22].

The aim of the present study was to assess the impact of hospitalisation on patients with IBD, particularly in terms of COVID-19 infection risk. Moreover, we investigated the levels of anxiety related to hospitalisation and their impact on disease relapse.

## 2. Materials and Methods

### 2.1. Patients and Study Protocol

At the end of first wave of the COVID-19 pandemic in Italy, in June and July 2020, we conducted a survey including consecutive IBD patients treated with biologics at three Italian referral centres (Pisa, Padua and Massa), referring to the lockdown period (11 March–4 May). We included consecutive adult patients treated with biologics, administered via i.v. (infliximab and vedolizumab) or s.c. (adalimumab, golimumab and ustekinumab). All patients included must be in clinical and biochemical (faecal calprotectin levels < 150 mg/kg [23]) remission at the time of the beginning of lockdown related to the first wave of COVID-19 pandemic. Clinical remission was defined as a Partial Mayo Score (PMS) < 2 for patients with ulcerative colitis, and as a Harvey–Bradshaw Index (HBI) < 5 for patients with Crohn’s disease. Patients experiencing a disease flare during these months, defined as an increase of 2 points of PMS or 3 points of HBI, as well as those with increased levels of faecal calprotectin (>150 mg/kg) were recorded during follow up-visits. 

Patients underwent the normally scheduled clinical visits every 8–12 weeks. Those treated with s.c. drugs were visited at home by using phone or video calls. Patients treated with i.v. drugs were visited at hospital the day of drug infusion, but in case of need (i.e., suspected COVID-19 symptoms or other reasons), a telemedicine visit was performed even in this population. At each visit, patients were asked to report if suspected symptoms for COVID-19 infection (such as cough, dyspnoea, malaise, dysgeusia, anosmia, fever > 38 °C) appeared, and even contacts with people affected by these symptoms were investigated.

**Figure 1:**
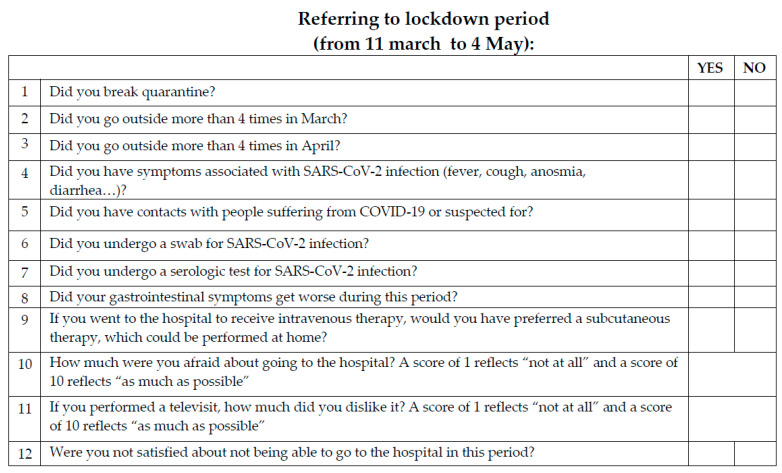
The 12 COVID-related additional questions administered to patients.

We administered to all patients the Hospital Anxiety and Depression Scale (HADS) questionnaire [24] and other 12 questions (Figure 1), specifically related to COVID-19 and its implications (quarantine, telemedicine, etc.), asking them to evaluate the period of the Italian lockdown (11 March–4 May). Thus, we investigated whether the lockdown situation had an impact on the perception of anxiety related to IBD treatment and to the need to move to high-risk places (i.e., hospitals or infusion centres). Regarding the HADS questionnaire, normal scores for anxiety or depression were defined by a score less than 8; a subscore from 8 to 10 was defined as borderline abnormal, and a score from 11 to 21 as abnormal [24]. In order to explore only the impact of the anxiety related to IBD and COVID, we excluded from the study patients with other comorbidities, immune-related (such as psoriasis, arthritis, etc.) or non-immune-related (such as a known metabolic, cardiovascular, or psychiatric disease, as well as the use of drugs that interfere with the nervous system for any reason). Furthermore, we excluded from the study pregnant women and patients who refused to give informed consent to participate to the study.

### 2.2. Statistical Analysis

Continuous variables are reported as mean ± standard deviation, while nominal variables are expressed as absolute number (percentage). Group differences in continuous and nominal variables were tested by Kruskal–Wallis test and Fisher exact test, respectively. For the items 10 and 11 of the COVID-related questionnaire, a score greater than 5 out of a 10-point scale was considered a positive response. Statistical analysis was performed using JMP Pro 14.3.0 software (SAS Institute Inc., Cary, NC, USA). A two-sided *p* < 0.05 was considered statistically significant.

### 2.3. Ethical Considerations

This study was conducted in full compliance with the Declaration of Helsinki. All patients signed an informed consent form to participate to the study and to the publication of the results; all questionnaires were treated anonymously. The present study was approved by local Ethics Committees.

## 3. Results

A total of 189 IBD patients were recruited. Patients’ characteristics are described in Table 1. Among them, 112 (59.3%) patients were treated at the hospital with i.v. biological drugs and 77 (40.7%) were treated at home with s.c. ones. Age (46 ± 15 years and 42 ± 14 years, respectively; *p* = 0.07) and sex distribution (49 (43.8%) and 34 (44.2%), respectively; *p* > 0.99) were similar between the two groups, whereas the prevalence of ulcerative colitis was significantly higher in those treated with i.v. drugs (50.0%) compared with those treated with s.c. drugs (22.1%; *p* = 0.0001).

**Figure 2:**
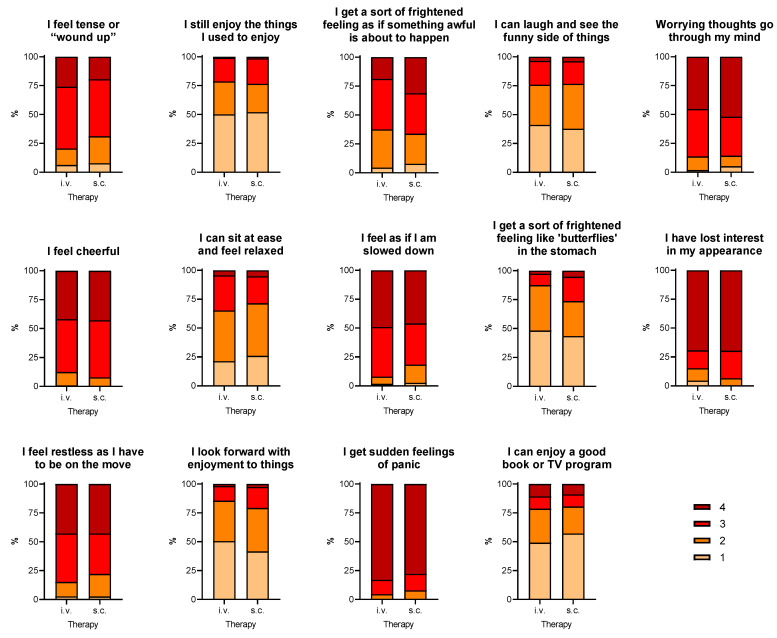
Responses to the HADS questionnaire in IBD patients treated with i.v. or s.c. biological therapy. i.v., intravenously; s.c., subcutaneously.

Among patients who needed access to the hospital, only one out of four would have preferred to be treated at home with s.c. therapy and was afraid of being infected in the hospital. IBD symptoms such as diarrhoea mildly worsened in 10 (8.9%) patients treated with i.v. drugs and 4 (5.2%) patients treated with s.c. drugs (*p* = 0.41), but the increase in PMS or in HBI was not sufficient to define a disease relapse in any patient (hospitalized vs. non-hospitalized, *p* = ns). Moreover, the two groups of patients had similar scores in the 14 single items of the HADS questionnaire (*p* > 0.10 for all) (Figure 2). The total HADS score obtained by the sum of all items was also almost identical between groups (37.1 ± 2.8 vs. 37.2 ± 2.8; *p* = 0.98). The two groups of patients had similar scores in the 14 single items of the HADS questionnaire (*p* > 0.10 for all) (Figure 2). The total HADS score obtained by the sum of all items was also almost identical between groups (37.1 ± 2.8 vs. 37.2 ± 2.8; *p* = 0.98).

**Figure 3:**
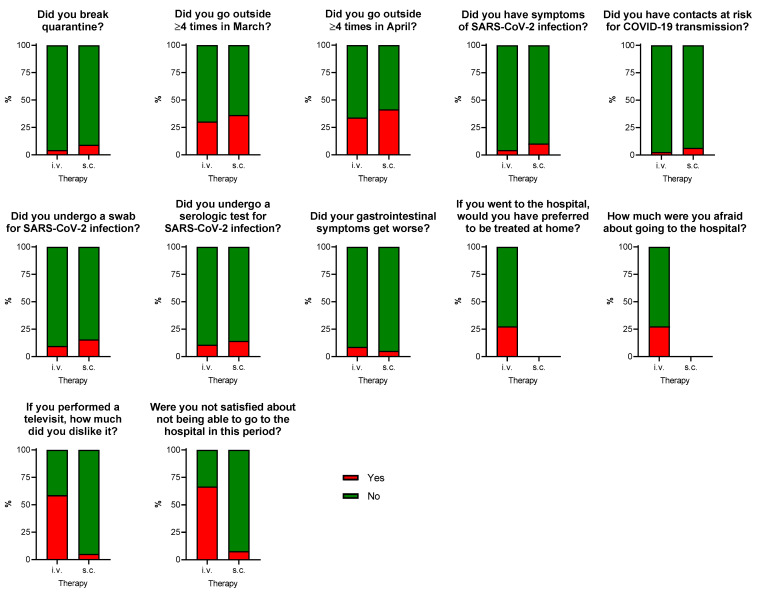
Responses to COVID-related additional questions in IBD patients treated with i.v. or s.c. biological therapy. A positive response indicates a score > 5 for the questions “How much were you afraid about going to the hospital?” and “If you performed a televisit, how much did you dislike it?”.

Among COVID-related questions (Figure 3), most (93.7%) participants declared they had strictly followed the quarantine rules over the lockdown period, going outside no more than once a week (four times each month) in March and April 2020. Thirteen (6.9%) patients had symptoms compatible with SARS-CoV-2 infection and eight (4.3%) had strict contacts with people with typical symptoms, without group differences (all *p* > 0.05). Thirty-seven (19.5%) patients underwent at least a diagnostic test for SARS-CoV-2 infection (molecular swab and/or serologic testing) with negative results. IBD symptoms such as diarrhoea mildly worsened in 10 (8.9%) patients treated with i.v. drugs and 4 (5.2%) patients treated with s.c. drugs (*p* = 0.41), but the increase in PMS or in HBI was not sufficient to define a disease relapse. Among patients who needed an access to the hospital, only one out of four would have preferred to be treated at home with s.c. therapy and was afraid to be infected in the hospital.

Most of the patients who received medical counselling via phone or video calls in the same period were satisfied with the visit (88.3%) and were satisfied not to go to the hospital (82.6%). However, in the subgroup of patients treated with i.v. drugs who also received medical counselling via telephone (*n* = 17), the rates of satisfaction with telemedicine (58.8%) and the lack of in-person care (33.3%) were significantly lower compared with those treated with s.c. drugs (94.8% and 92.2%, respectively; both *p* < 0.0005). 

## 4. Discussion

The COVID-19 pandemic is having a huge impact on daily habits worldwide and even the access to hospitals has been extensively revised in comparison with prepandemic era [21,22,25]. Patients with chronic diseases, such as IBD, had to adapt to this new situation, where the risk of contracting SARS-CoV2 infection when leaving home is counterbalanced by the necessity of maintaining current treatment, which in some cases requires hospitalisation. For these reasons, some authors suggested the need to convert i.v. therapies to s.c. ones, in order to reduce the risk of infection and the fear related to movements outside the home [19,20].

The present study corroborates the evidence that patients with IBD treated with biologics do not have an increased risk of COVID-19. Indeed, only a small percentage of patients (6.9%) included in the study had symptoms compatible with SARS-CoV-2 infection and just eight (4.3%) had strict contact with people with typical symptoms, without group differences between the hospitalized patients and those who could remain at home. Moreover, the 19.5% of patients included in the study underwent at least one diagnostic test for SARS-CoV-2 infection (molecular swab and/or serologic testing), and all results were negative in both hospitalized and non-hospitalized patients. The latter finding is particularly relevant since suggests that hospitalisation for drug infusion does not represent a risk factor for infection spread and provides evidence for the current recommendations suggesting to continue therapies at infusion centres, without the need of converting i.v. biologics to s.c. drugs [18]. With regard for the general risk of infection in IBD population, data collected in China showed that there were no patients with IBD infected with SARS-CoV-2 in the seven largest IBD referral centres, with more than 200,000 patients evaluated since the beginning of the pandemic [26]. Pooled data confirm that COVID-19 does not occur more frequently in patients with IBD than in the general population [27,28]. Since the worst forms of COVID-19 develop as the result of an uncontrolled and overactive immune response against the virus [29], the immunosuppressed state due to biological therapy may actually protect patients from it [30]. Indeed, therapies with anti-TNF were associated with better outcomes of COVID-19 in IBD patients who contracted SARS-CoV-2 infection [31] and active phases of IBD are associated with a more severe course of COVID-19 [32]. A large multicentre study in patients with rheumatic diseases confirmed these findings, since anti-TNF therapies were associated with decreased odds of hospitalisation for COVID-19, while neither the exposure to conventional disease-modifying antirheumatic drugs nor non-steroidal anti-inflammatory drugs were associated with increased odds of hospitalisation [33]. Accordingly, Veenstra et al. [34] displayed the same results in a cohort of patients with different immune-mediated inflammatory diseases receiving immunosuppressive therapies.

Notably, the present study shows that our patients with IBD were not particularly afraid of SARS-CoV-2 infection, since the levels of anxiety detected by HADS questionnaire are similar in patients who could remain at home and in those who needed to go to the hospital. It is worthy of note that anxiety and stressor events have been associated with an increased risk of disease relapse [7,8,9], In particular, a prospective study by Wintjens et al. [9] showed that the occurrence of life events in the preceding three months was positively associated with IBD flares. A recent review by Sun et al. [8] confirmed these findings, demonstrating that psychological stress could induce IBD relapses both in adult and in paediatric patients by several mechanisms, such as impaired intestinal barrier function, disturbance of the gut microbiota, intestinal dysmotility, and immune and neuroendocrine dysfunctions. In the present study, IBD symptoms mildly worsened only in ten (8.9%) patients treated with i.v. drugs and in four (5.2%) patients treated with s.c. ones (*p* = 0.41), but the increase in PMS or in HBI was not sufficient to define a disease relapse. Interestingly, we report that the anxiety scores were not different in the two groups, both in the 14 single items of the HADS questionnaire and in the total HADS score obtained by the sum of all items. Unfortunately, since we did not perform a prepandemic HADS questionnaire we could not compare HADS responses before and after the development of the pandemic. The mean levels of HADS obtained in both cohorts could be considered high, in accordance with the literature [35]. However, if the pandemic had had a significant impact on anxiety levels in our patients, we would have expected that patients who could stay at home would have had lower levels than those who needed to go to the hospital. Conversely, our data showing similar levels on the HADS questionnaire in both cohorts could be surprising, since several studies conducted even in heathy subjects showed how the COVID-19 pandemic significantly increased the levels of stress and anxiety [36,37,38]. On the other hand, two studies evaluating patients with chronic diseases (Parkinson’s disease [39] and multiple sclerosis [40]) are in line with our findings, showing how the COVID-19 pandemic did not significantly increase the HADS scores. Probably, the attitude to living in contact with hospitals and other patients with different diseases reduced the fear and the anxiety of developing new pathologies, such as COVID. This could be partially related even to the easier contact of these patients, as compared with general population, with health-care providers, who could reassure the patients about the implications of their disease and the possible SARS-CoV-2 infection. However, about one patient out of four would have preferred to be treated at home with s.c. therapy instead of going to the hospital, but we should consider this finding as acceptable during the first wave of COVID-19 pandemic, where no patient had certainties about this disease. To confirm the almost good confidence of IBD patients, it is worthy of note that none of the patients included in this study missed any appointment during lockdown period, and this is particularly important in patients who needed to go to the hospital. Indeed, considering all patients treated with biologics, we did not observe a reduction in medical activity compared with the prelockdown period, as we recently reported in adult patients [41] in contrast with the survey by Khan et al. [42]; our findings were confirmed in a large Italian survey even in paediatric patients [43]. We acknowledge that the HADS questionnaire displays some limits in evaluating a specific situation such as this. However, it is easy-to-use for both patients and physicians and there are several papers which validated it and corroborated its use. Moreover, we administered the 12-item specific questionnaire aimed at investigating the COVID-related condition.

According to our data regarding SARS-CoV-2 infection rates in our population, the similarity of anxiety levels between patients treated with i.v. therapies and those treated with s.c. drugs support the decisions taken by the main scientific societies, which presently recommend to maintain the current biological treatment even during the lockdown period, avoiding switching from i.v. to s.c. therapies [18].

Finally, the present study provides another important finding regarding the difference in terms of acceptability of telemedicine by patients treated with i.v. therapies as compared to those treated with s.c. ones. Telemedicine has become an important tool in managing patients with different chronic diseases in recent years, and its use was proposed before COVID-19 pandemic since it could reduce the hospitalisations [44,45]. As expected, international scientific societies suggested implementing telemedicine in order to monitor IBD patients during lockdown [18]. Globally, patients with IBD showed a positive attitude regarding telemedicine consultancies [21,46,47], probably because their age is relatively low and they are aware about using technological tools. To confirm this, a survey conducted in patients with different chronic diseases during the COVID-19 pandemic showed that patients with IBD (especially the younger ones) were the most inclined to accept telemedicine and showed great satisfaction with it [47]. Our results are in line with previous findings, since the rate of satisfaction about remote visits was extremely high (88.3%), particularly because patients reported that they were satisfied about not having to go to the hospital (82.6%). Interestingly, the rates of satisfaction were higher in patients treated with s.c. drugs as compared with those treated with i.v. ones. In our opinion, a possible explanation could be that patients treated with s.c. drugs are more accustomed to a quick contact with the doctor, aimed only at receiving the drug which is administered independently at home. Conversely, patients treated with i.v. drugs are used to staying in hospital for hours during the infusions, having prolonged in-person contact with nurses and doctors, and a remote visit is very different from their standard visits. However, to the best of our knowledge, this is the first report of a difference in the perception of telemedicine in specific categories of IBD patients, and this should be taken into consideration in order to personalize treatment.

Our study has some limitations. Firstly, this survey was conducted at the end of the first wave, when patients were more aware about COVID-19 and its implications. Although we promoted this survey just a few days after the end of the lockdown period, and we were extremely clear in asking our patients to refer the answers to the lockdown period, a prospective study would be more convincing in evaluating anxiety levels in these specific moments. Another important limitation is related to sample size, which should be greater in order to confirm some findings of our results, particularly the differences in the perception of telemedicine. Future studies with this specific purpose should be performed. We even acknowledge thatSARS-CoV-2 PCR testing in all patients should be more appropriate to determine the real rate of COVID-19 in the patients’ cohort, but the present study was not designed with this specific aim. Lastly, the observation period is somewhat too short to properly evaluate the relapse rate in a cohort of patients with IBD treated with biologics. However, if the need to go to the hospital had increased anxiety levels, we would expect a higher rate of disease relapse in patients treated with i.v. drugs, even in a short-term follow-up.

On the other hand, our study has the important strength of the methodology: the choice of selecting only patients in clinical and biochemical remission without significant comorbidities allows us to be more confident about our results, excluding several confounding factors affecting anxiety levels. Moreover, this is the first report of a difference in satisfaction about telemedicine in a specific cohort of patients with IBD, which could suggest the personalization of the choice of performing remote visits, if further confirmed.

## 5. Conclusions

To conclude, our results suggest that there is no need to convert patients from i.v. to s.c. therapy during COVID-19 outbreaks, since the risk of infection and its transmission is not increased. Moreover, anxiety levels are similar in both groups, emphasizing that hospitalisation seems not to affect the psychological status of the patients, thus not increasing the relapse rate. Interestingly, we even showed that patients used to coming to the hospital have more need for in-person contact than patients used to being treated at home, suggesting that the choice of telemedicine should be personalized.

## Figures and Tables

**Table 1:** Patients’ characteristics.

Number of patients, *n*	189
Female, *n* (%)	83 (44)
Age, years, median	45 (19–76)
Crohn’s disease, *n* (%)	116 (61)
Drug	
Infliximab, *n* (%)	71 (38)
Adalimumab, *n* (%)	41 (23)
Golimumab, *n* (%)	7 (4)
Vedolizumab, *n* (%)	39 (21)
Ustekinumab, *n* (%)	29 (15)

## Data Availability

Data are available upon request to Corresponding Author.
